# The Relationship between Regular Sports Participation and Vigilance in Male and Female Adolescents

**DOI:** 10.1371/journal.pone.0123898

**Published:** 2015-04-07

**Authors:** Rafael Ballester, Florentino Huertas, Francisco Javier Yuste, Francesc Llorens, Daniel Sanabria

**Affiliations:** 1 Department of Physical Activity and Sport Sciences, Universidad Católica de Valencia “San Vicente Mártir”, Valencia, Spain; 2 Mind, Brain and Behavior Research Center, Universidad de Granada, Granada, Spain; 3 Department of Experimental Psychology, Universidad de Granada, Granada, Spain; Defence Science & Technology Organisation, AUSTRALIA

## Abstract

The present study investigated the relationship between regular sport participation (soccer) and vigilance performance. Two groups of male and female adolescents differentiated in terms of their sport participation (athletes, n = 39, and non-athletes, n = 36) took part in the study. In one session, participants performed the Leger Multi-stage fitness test to estimate their aerobic fitness level. In the other session, participants completed the Psychomotor Vigilance Task (PVT) to evaluate their vigilance performance. Perceived arousal prior to the task and motivation toward the task were also measured in the PVT session. The results revealed that athletes had better cardiovascular fitness and showed better performance in the PVT. However, correlation analyses did not show any significant relationship between cardiovascular fitness and performance in the PVT. Athletes showed larger scores in motivation and perceived arousal measures with respect to non-athletes, although, once again, these variables were not correlated with PVT performance. Gender differences were observed only in the Leger test, with males showing greater fitness level than females. The major outcome of this research points to a positive relationship between regular sport participation and vigilance during adolescence. This relationship did not seem to be influenced by gender, perceived arousal, motivation toward the task or cardiovascular fitness. We discuss our results in terms of the different hypotheses put forward in the literature to explain the relationship between physical activity and cognitive functioning.

## Introduction

A positive relationship has been established between physical activity, brain functioning and cognitive performance in children [1,2]. For these reasons, the last two decades have seen a rapid growth of interest in elucidating the relationship between physical activity and the development of cognitive functions. Recently, an extensive literature review has investigated the psychological and social benefits of regular participation in sports in children and adolescents [3]. That study concluded that team sport participation is related with improved psychosocial health outcomes compared to individual physical and non-physical activities. However, few studies have investigated the relationship between cognitive processing and regular sport participation during adolescence. In the present study we focus on soccer (football) since it is probably the most popular sport in the world.

Regarding physical benefits, research has found that soccer practice enhances musculoskeletal, metabolic and cardiovascular adaptations in adults [4]. Accordingly, the few studies that have addressed this topic during adolescence have reported positive effects of regular soccer practice on different physical parameters such as sprinting speed, agility, isometric maximal strength, explosive strength, and cardiorespiratory fitness, both at recreational [5] and elite level [6].

Soccer participation has also been related to improved perceptual-motor skills. Studies have shown that young adult soccer players outperformed non-athletes and/or less skilled soccer players in different measures of attentional orienting and executive functioning [7–9]. Researchers have also shown a positive relationship between soccer participation and performance in specific tests of visual function [10], and general tests of perceptual and motor performance during adolescence [11]. However, to the best of our knowledge, no study has examined the relationship between regular sport participation and vigilance performance in adolescents.

Vigilance refers here to the capacity to maintain attention over time and the ability to respond appropriately to relevant stimuli [12]. Low levels of vigilance result in slow responses and even failures to respond to target stimuli. Another key finding in vigilance research is that performance in attentional tasks declines over time, a result known as the vigilance decrement. This performance decrement over time has been related to a continuous depletion of attentional resources [13,14].

Sufficient levels of vigilance are necessary in many day-life activities, such as attending to academic lessons at school. In effect, a positive relation has been found between vigilance and academic achievement in adolescents [15,16]. Therefore, investigation of the variables that might contribute to the capacity of maintaining an optimal level of vigilance during adolescence is highly relevant.

Both vigilance performance [17] and cardiovascular fitness [18] seem to be influenced by gender. However, while some studies have investigated the physical benefits of regular soccer participation in male adolescents [5,6], no study has investigated this relationship in female adolescents. In the same line, no previous study has considered the potential role of gender in the association between sport participation and vigilance. Thus, the inclusion of female adolescents in our study represents a step forward from previous research.

We also explored the relationship between cardiovascular fitness and vigilance performance in adolescents. To the best of our knowledge, there are no studies that have investigated these factors directly, with some scarce evidence in children [19] and young adults [20]. Pontifex et al. [19] showed less errors of omission in a flanker (conflict) task in highly fit 9–10 year old children than in low-fit children, a result that was taken as evidence of superior sustained capacities in the highly fit group, even though the flanker task was not (at least initially) designed to measure sustained attention or vigilance. Meanwhile, Luque-Casado et al. [20] showed better performance in the Psychomotor Vigilance Task (PVT), showing overall faster reaction times (RT), in high-fit young adults than in their low-fit counterparts.

Together with cardiovascular fitness, we also investigated the potential influence of variables that have been previously related to vigilance performance, such as perceived activation/arousal before the task [21] and motivation toward the task [22, 23].

In sum, our approach is novel and differs from the majority of studies on this research topic. The lack of previous evidence regarding the links between regular sport participation and vigilance performance during adolescence motivated the present research. On the basis of previous research, we expected athletes, both male and females, to outperform non-athletes in both cardiovascular fitness and vigilance performance.

## Method

### Ethics Statement

The study protocols and procedures were approved by the *Research Institute of Sport Sciences* of the *Catholic University of Valencia*. The study was performed in full compliance with the Declaration of Helsinki 1964. All participants were given verbal and written information about the experiment. They were also informed about their right to leave the experiment at any time. All subjects participated in the study under the written consent of their parents and club or school.

### Participants

Seventy-five adolescents (see Table 1) were recruited to participate in this study. Thirty-nine adolescent (15 females) soccer players from a Spanish League junior team were selected to be part of the athletes group. Participants of this group attended specific training sessions three times per week and a competition match on weekends (5 hours per week of systematic soccer practice). Moreover, all of them reported more than five years of systematic and deliberate soccer practice. Thirty-six students (18 females) from a local public school were selected to be part of the non-athletes group. The participants in the non-athletes group met the inclusion criteria of not reporting sport experience or systematic sport practice out of school (less than 2 hours per week of sport participation out of school). None of the participants had an individual education plan or accommodations to receive direct or indirect special education services (e.g., attention deficit disorder; cognitive or physical disability). Note that an a priori power analysis was conducted to determine the minimum sample size required for a power level of. 80. This analysis was based on data from a previous study by Luque-Casado et al. [20] who compared performance in the PVT of a group of young cyclists and triathletes (high-fit) to that of a group of non-athletes (low-fit) young adults. This analysis gave an outcome of 22 participants per group.

**Table 1:** Mean (SD) of participants’ demographic, fitness, PVT results and scores in the visual analogue scales.

***Demographic and anthropometrical characteristics***				
Group	Non-athletes(n = 36)		Athletes(n = 39)	
Gender	Male(n = 18)	Female(n = 18)	Male(n = 24)	Female(n = 15)
Age (years)	13.9 (0.6)	13.7 (0.6)	13.4 (0.3)	13.9 (0.6)
BodyMass Index (kg/m^2^)	19.1 (2.7)	21.7 (3.2)	20.3 (1.7)	20.5 (2.0)
***Cardiovascular fitness measures***				
Resting HR (bpm)	79 (9) *	80 (10)	67 (8)	64 (6)
Maximal HR (bpm)	203 (7)	201 (8)	203 (6)	201 (4)
Time to Exhaustion (sec)	353 (59) * †	228 (82)	544 (94)	477 (65)
HRR_60_ index (%)	19.8(5.8) * †	16.9 (6.9)	35.6 (9.8)	17.9 (6.3)
HRR_180_ index (%)	32.7 (5.9) * †	30.6 (8.5)	43.4 (4.5)	36.9 (4.3)
HRR_300_ index (%)	36.2 (4.2) * †	32.3 (6.9)	47.4 (4.8)	40.5 (3.6)
***Psychomotor Vigilance Task***				
Mean RT (ms)	355 (72) *	369 (73)	309 (53)	302 (26)
Lapses (number)	5.9 (5.7) *	6.1 (5.4)	2.0 (1.9)	2.5 (1.7)
***Visual analogue scales***				
Activation prior to the task	67(30) *	61 (31)	73 (28)	82 (23)
Motivation toward the task	73 (23) *	73 (25)	79 (16)	88 (12)
PVT cognitive workload	27 (22)	38(28)	37 (28)	48 (27)

* Indicates significant differences between groups (p < 0.05).

† Indicates gender-significant differences (p < 0.05).

### Apparatus, materials and procedure

Participants were evaluated on two separate occasions with a minimum interval of 2 days and a maximum interval of 7 days, at the same time of the day. On the *Psychomotor Vigilance task session*, participants were fitted with a Polar RS800CX HR monitor (Polar Electro Ltd., Kempele, Finland). Subsequently, they rested for five minutes in a seated position to record the resting heart rate (HR). Then, they rated on a 0–100 visual analogue scale (a modified version of a scale developed by Monk, [24]) their subjective activation and their motivation toward the task. Subsequently, all participants completed the PVT (see details below) using the same laptop model, an HP Pavilion g series (15’6-inch color screen), running the E-Prime software (Psychology Software Tools, Pittsburgh, PA, USA) that controlled the presentation of stimuli, timing operation, and collection of responses. Participants performed the PVT sitting on a chair at 60 cm from the computer monitor. The resting HR recording and the PVT were completed in a dimly illuminated and noise-reduced room. After the PVT was finished, participants rated the perception of cognitive workload of the PVT in the 0–100 visual analogue scale.

In the *Fitness Assessment Session*, a brief anthropometric study was performed on each participant to calculate the body mass index (BMI; i.e., weight divided by the square of the height). Participants were again fitted with the HR monitor. Then, in groups of eight participants, the *Léger Multi-stage fitness test* [25] was performed (see details below). This test or its adaptations is one of the most common assessment procedures used for measuring cardiorespiratory fitness in studies involving young participants [26].

The *Psychomotor Vigilance task* and *Fitness assessment* sessions were completed in counterbalanced order. At the end of the last session, participants were debriefed on the purposes of the study and given an explanation of their cardiovascular fitness with easily understandable data.

### Psychomotor Vigilance Task

Vigilance was measured by means of the PVT [27]. The PVT requires participants to respond, as rapidly as possible, to a visual stimulus that appears on the screen. The PVT is a simple and reliable task to measure vigilance given the monotonous, repetitive, and unpredictable nature of the target onset [28]. This task can range from as short as 8 minutes for attentional stores to be depleted sufficiently to observe failures in vigilance [29], and even less (5 minutes) in children [30].

**Fig 1:**
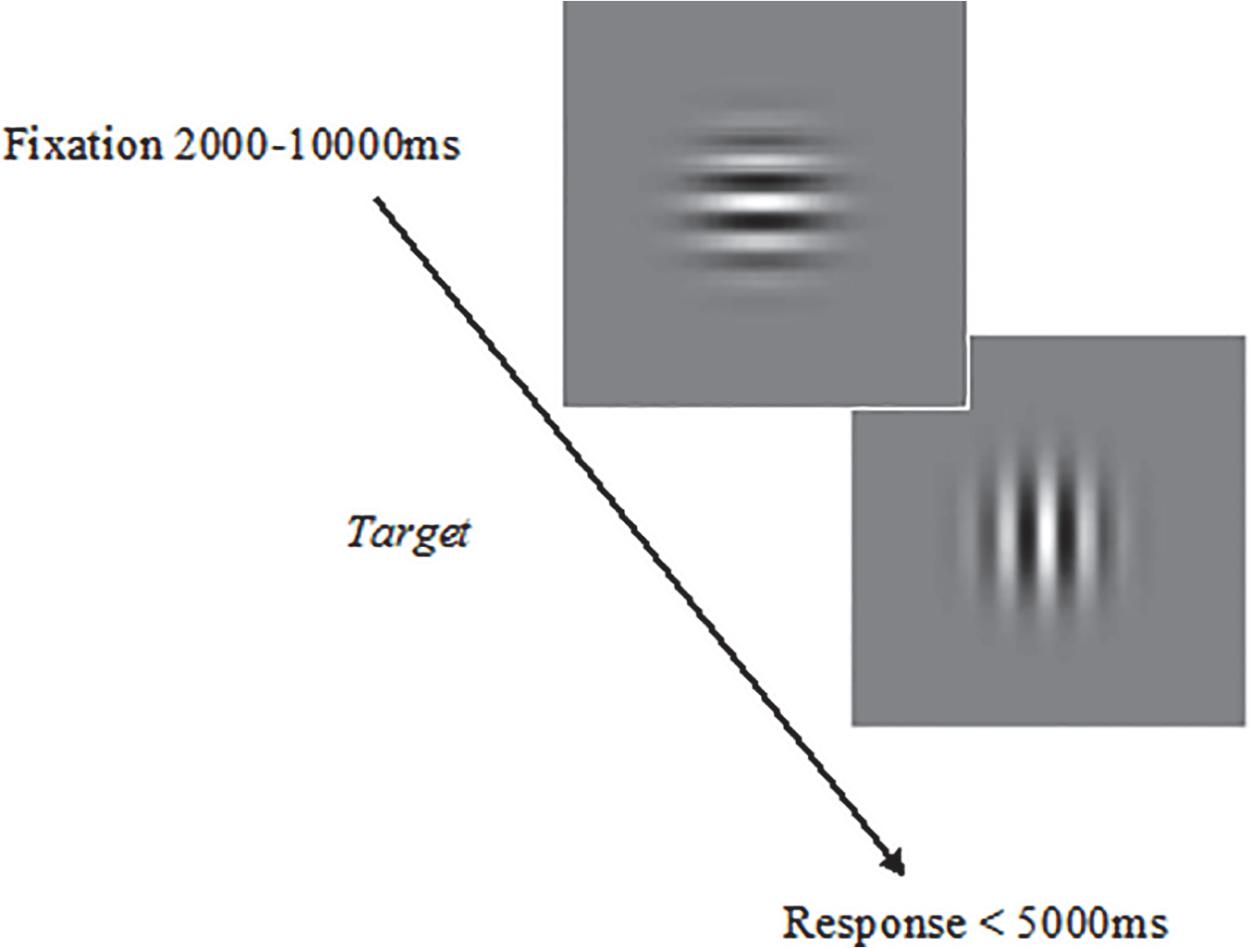
Schematic view of the temporal course of a trial sequence in the PVT (from top left to bottom right).

During the task, on each trial appeared a Gabor patch (4.20° x 4.20°) with a horizontal orientation at the center of the screen in a grey background. Later, at a random time interval (between 2000 and 10000 ms), the lines abruptly changed their orientation to vertical (Fig 1). The participants were instructed to respond to this change as fast as possible by pressing the space bar on the laptop keyboard with the index finger of their dominant hand. Feedback of the response time was displayed on the screen after each trial during 300 ms interval before the next trial began. If a response had not been made within 5000 ms, the message ‘‘You did not answer” appeared on the screen and the next trial began. The task lasted for 9 minutes without interruptions.

### Léger Multi-stage fitness test

This test was originally designed to determine the maximal aerobic power of schoolchildren and healthy adults [25]. Participants run back and forth on a 20-m course and had to reach the 20-m line at an initial speed of 8.5 km/h which increased progressively (0.5 km/h every minute) in accordance with a pace dictated by a prerecorded tape. Several shuttle runs of 20m made up each stage, with each stage lasting for one minute. As the test proceeded, the number of shuttle runs increased in each stage and the participants were instructed to keep the pace with the acoustic signal for as long as possible. The test finished when the participants acknowledged voluntary exhaustion or were not able to follow the pace during two consecutive acoustic signals. The maximal HR (HR_max_) of each participant was annotated right after the end of the test. The last on-time lap completed was used to define the Time to Exhaustion (TTE) that was used as our main index of the cardiovascular fitness level of our participants. Additional indices of cardiovascular fitness based on participants’ HR were also measured. After completing the physical test, participants stayed in the upright seated position (inactive recovery, [31]) during five minutes to calculate the HR Recovery index (HRR), measured as the percentage of reduction of the HR with regard to the HR_max_ following 60 seconds of recovery (HRR_60_), following 180 seconds of recovery (HRR_180_) and following 300 seconds of recovery (HRR_300_). Post-exercise HRR has been reported to be an important index of exercise endurance capacity and individual cardiovascular fitness [32]. HR was continuously monitored throughout the session using a sampling rate of 1 Hz.

### Design and Statistical analysis

Analyses of variance (ANOVAs) with the between participants factors of gender (male, female) and sport participation (athletes vs. non-athletes) were used to analyze the physiological and behavioral data. Participants’ mean reaction time (RT) and lapses (number of trials with RT above 500 ms; cf. [28]) were introduced in ANOVAs with the between participants factors of gender (male, female) and sport participation (athletes vs. non-athletes) and the within participants factor of time on task (block 1, 2 and 3). Note that data from the 9 minutes of the PVT were divided in three blocks of 3 minutes each to investigate the temporal course of participants’ RTs and lapses in order to explore the so-called vigilance decrement throughout the task. For the PVT analysis, trials with RTs below 100 ms (1.0%) were considered anticipations [33] and therefore discarded from the analysis. In the analysis of the PVT data, the Greenhouse–Geisser correction was applied when sphericity was violated [34], and corrected probability values are reported. Tukey HSD posthoc tests were used to analyze further significant main effects (with more than two experimental conditions) and interactions.

In order to investigate the relationship between vigilance performance and cardiovascular fitness we performed Pearson correlations between data from the PVT (overall mean RT and number of lapses) and TTE, our main index of fitness, within each group of participants (39 athletes and 36 non-athletes). Similar correlation analyses were performed between data from the PVT and scores in the scales of motivation toward the task, perceived arousal prior the task and perception of the PVT cognitive workload.

## Results

### Physiological measures

The ANOVA with participants’ *TTE* in the *Léger Multi-stage fitness test* revealed main effects of sport participation, *F*(1,71) = 144.88, *p* < .001, η^2^_partial_ = .67 and gender, *F*(1,71) = 27.65, *p* < .001, η^2^_partial_ = .28. Athletes outperformed non-athletes and boys showed greater *TTE* values than girls (see Table 1). The interaction between sport participation and gender was not significant, *F*(1,71) = 2.56, *p* = .11.

The two groups were not different in *BMI*, *F < 1*, *p* = .98. In contrast, the analyses revealed that athletes had lower resting HR than non-athletes, *HR*_*rest*_, *F*(1,71) = 52.23, *p* < .001, η^2^_partial_ *=* .42. Importantly, the non-significant difference between groups in HR_max_, *F < 1*, *p* = .78, suggests that participants in both groups gave a maximal effort in the physical test.

Athletes recovered their heart rate faster than non-athletes throughout all after exercise resting times: *%HRR*_*60*_, *F*(1,71) = 21.97, *p*<.001, η^2^_partial_ = .24, *%HRR*_*180*_, *F*(1,71) = 36.25, *p* < .001, η^2^_partial_ *=* .34, *%HRR*_*300*_, *F*(1,71) = 67.31, *p* < .001, η^2^_partial_ *=* .49.

Regarding gender, the analyses revealed differences between males and females in *%HRR*_*60*_, *F*(1,71) = 33.40, *p* < .001, η^2^_partial_ = .32, *%HRR*_*180*_, *F*(1,71) = 9.26, *p* = .003, η^2^_partial_ *=* .34, and *%HRR*_*300*_, *F*(1,71) = 20.76, *p* < .001, η^2^_partial_ *=* .23, with lower values for females than for males (see Table 1). No differences were found in *HR*_*rest*_ and HR_max_ (both *Fs<1*). The interaction between sport participation and gender was significant for *%HRR*_*60*_, *F*(1,71) = 17.13, *p* < .001, η^2^_partial_ = .19. Tukey HSD post-hoc tests showed that male athletes showed better *%HRR*_*60*_ than male non-soccer players, *p* < .001, without differences in *%HRR*_*60*_ between females, *p =* .*98*. The interaction between sport participation and gender was not statistically significant either for *%HRR*_*180*_, *F*(1,71) = 2.51, *p* = .12, or *%HRR*_*300*_, *F*(1,71) = 1.56, *p* < .22. None of the rest of the terms in the ANOVAs were significant (all *Fs < 1*).

### Motivation, Activation and Subjective Workload

The analyses revealed a significant main effect of sport participation in terms of *Activation prior to the Task*, *F*(1,71) = 4.74, *p* = .03, *η* ^2^_partial_ = .06 and *Motivation toward the Task*, *F*(1,71) = 4.94, *p* = .03, *η* ^2^_partial_ = .06. Athletes showed greater values than non-athletes in these subjective measures. However, no significant differences between groups were obtained in *Perception of the PVT cognitive workload*, *F*(1,71) = 2.56, *p* = .11. No gender differences were found in *Activation prior to the Task*, *Motivation toward the Task* or *Perception of the PVT cognitive workload* (*all* Fs < 1). The interaction between sport participation and gender was not significant for any of the variables (all *F*s < 1*)*.

### Vigilance performance

The analysis of the participants’ mean RTs revealed significant main effects of sport participation, *F*(1,71) = 17.27, *p* < .001, η^2^_partial_ = .19 (see Table 1) and time on task, *F*(1.50,106.67) = 5.84, *p* = .003, η^2^_partial_ = .08. Athletes were faster than non-athletes. Regarding the main effect of time on task, Tukey HSD post-hoc tests showed significant differences between block 1 (317 ms) and block 3 (353 ms), *p*<.001. Neither the difference between block 1 and block 2 (330 ms), *p* = .36, nor that between block 2 and block 3, *p* = .05, were statistically significant. None of the remaining terms in the ANOVA reached statistical significance (all *F*s < 1).

**Fig 2:**
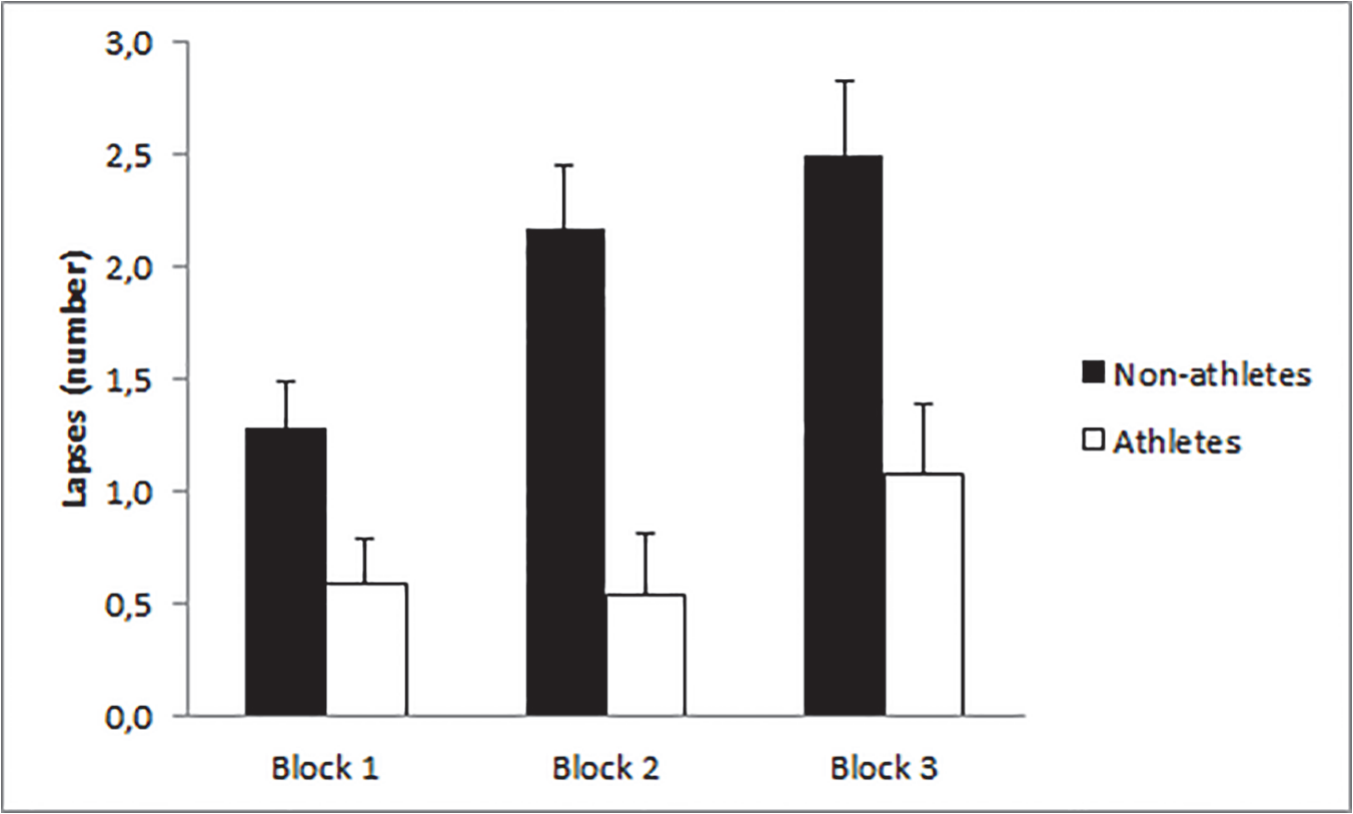
Lapses as a function of time on task and group. Number of lapses for the non-athletes and athletes group in each of the 3 blocks of the PVT. Bars represent standard errors of the mean. * p<.05

The analysis of the participants’ lapses revealed significant main effects of time on task, *F*(1.71,122) = 7.41, *p* < .001, η^2^_partial_ = .09, and sport participation, *F*(1,71) = 15.08, *p* < .001, η^2^_partial_ = .18. Interestingly enough, these main effects were better qualified by the significant interaction between time on task and sport participation, *F*(1.71,122) = 3.20, *p* = .04, η^2^_partial_ = .04 (Fig 2). Tukey HSD post-hoc test showed that athletes did not show significant difference in the number of lapses either between block 1 and block 2, *p* = .99, between block 1 and block 3, *p* = .53, or between block 2 and block 3, *p* = .41. In contrast, non-athletes showed a significant increase in the number of lapses from block 1 to block 2, *p* = .03. The difference between block 2 and block 3 was not significant, *p* = .87. None of the remaining terms in the ANOVAs were statistically significant (all *F*s < 1).

### Correlation analyses

The correlation analyses did not show any significant relationship between the PVT main dependent variables (overall mean RT and lapses) and the scores of *Activation prior to the task*, *Motivation toward the task* and *Perception of the PVT cognitive workload* in any of the groups (all *r* ^2^<.06 and all *p*s >. 2). Crucially, none of the correlations between the PVT main dependent variables and the main index of cardiovascular fitness (TTE) were significant (all *r* ^2^<.04, and all *p*s >. 27).

## Discussion

The main purpose of our research was to investigate the relationship between sport participation and vigilance performance during early adolescence, exploring the role of gender on this relationship. We were also interested in looking at the potential influence of cardiovascular fitness, motivation toward the task and subjective arousal on the relationship between sport participation and vigilance performance.

Our results showed that the level of cardiovascular fitness was significantly greater in athletes than in non-athletes. This result goes beyond previous research by showing superior physical fitness not only in male [5,6], but also in female adolescent soccer players compared to their age-matched counterparts. This difference in cardiovascular fitness between athletes and non-athletes was expected since fitness capacity is constantly stimulated in different sports, such as soccer, through training and competition [35].

Concerning performance in the PVT, we found no significant differences between males and females. This result is consistent with those of a study by Venker et al. [36] who also used the PVT to evaluate vigilance in children. While they found gender differences at the age of 6, with boys responding faster than girls, these differences disappeared progressively until performance was approximately equal by age 11. However, the results reported here seem to be inconsistent with those reported in a previous study by Beijamini et al. [17], who found faster RTs and fewer lapses in males than female adolescents of 13–16 years old in the PVT. On the other hand, other studies have shown that gender differences are present in young and older adults [37] indicating that differences may reemerge during adolescence. Given the lack of consistency in the literature mentioned above, further research is needed to clarify the role of gender in vigilance during adolescence. In any case, gender did not seem to influence the relationship between sport participation and vigilance performance.

Crucially, our results showed that athletes responded faster and with fewer lapses than non-athletes. In terms of lapses (RT>500 ms), athletes were also able to maintain a similar level of performance throughout the task. In contrast, non-athletes showed a significant increase in the number of lapses over the course of the PVT. These results suggest a positive relationship between regular sport participation and vigilance performance, which is not trivial, given the importance of maintaining a sufficient level of vigilance in many day-life activities during adolescence. Interestingly, the between-group difference in vigilance capacities did not seem to be influenced by motivation toward the task and/or perceived activation prior to the task, even though the two groups of participants significantly differed in these two measures.

When looking at the possible causal relations between physical activity and cognitive processing, previous research have shown that chronic aerobic exercise results in enhanced brain structure and functioning throughout the life span ([38], for a review). The “cardiovascular fitness hypothesis” [39] suggests that cardiovascular fitness is the physiological mediator that explains the cognitive benefits of physical activity. Nonetheless, the results of an extensive meta-regression [40] did not support that hypothesis and concluded that cardiovascular fitness was unrelated to cognitive performance among children and adolescents, suggesting that other variables than aerobic fitness may play an important role on the relationship between cognitive processing and physical activity. In fact, in the same line two results reported here are inconsistent with the “cardiovascular fitness hypothesis”: 1) the non-significant correlations between the indices of vigilance performance (overall RT and lapses) and the main index of physical fitness (TTE), and 2) the non significant differences in performance in the PVT between males and females, although they were considerably different in terms of cardiovascular fitness.

Any discussion regarding the lack of a significant relationship between vigilance performance and cardiovascular fitness in our participants should, however, should take into account the fact that the PVT, in contrast to the flanker task used by Pontifex et al. [19] and other vigilance tasks that involve response inhibition to unpredictable stimuli (e.g., the sustained attention to response task, SART, [41]) does not involve high executive demands. In that sense, the “selective improvement hypothesis” [42], which has complemented the “cardiovascular fitness hypothesis” suggests that gains in cardiorespiratory fitness, lead to selective, rather than generalized cognitive benefits, showing throughout studies the largest fitness-related improvements in tasks with substantial executive control demands and minor-to-no effects in tasks that do not require executive functioning [42–44]. Thus, one might argue that the lack of a significant relationship between cardiovascular fitness and the measures of cognitive processing used in our study could be due to the low executive demands of the PVT used here.

Interestingly, our findings seem to be consistent with the *“*cognitive component skills” theory [45,46], which highlights that athletes show enhanced cognitive performance in perceptual-cognitive measures outside of the sport context. According to Voss et al. [46], this hypothesis considers *“sport training as a medium for experience dependent brain plasticity*, *or cognitive training*, *that results in more efficient brain networks (both general and sport-specific)”* which would result in enhanced perceptual-cognitive skills. For instance, a recent study by Vänttinen et al. [11] with adolescents, showed greater performance in soccer players than in non-athletes in a simple RT task that lasted for less than 1 minute and had only five trials. Here, we replicated these results and went a step forward by showing superior vigilance performance in adolescents as a function of regular participation in soccer.

It is worth noting, however, that any potential explanation of the present results needs to take into consideration the nature vs. nurture debate, which is inherently linked to any cross-sectional study related to the topic of this research. Did our athletes start playing soccer because of their superior cognitive abilities to excel in the field, or did they develop and reinforce their cognitive skills through sport-dependent learning? This question cannot be unequivocally addressed without long-term follow-ups and longitudinal studies that track athletes at various levels in different sports, to investigate how cognitive abilities may differ as a function of sport-dependent experience and learning.

In relation to the sport-dependent learning argument, some authors have pointed out that demands of perceptual-cognitive skills required during sport training environment may lead to a reduction in the processing of task-irrelevant information and to an adaptation of the most frequent task demands [47]. This would increase the allocation of attentional resources to deal with unusual conditions [47]. In our study, we found that the overall RT and lapses were significantly lower (and more resistant to time on task effect in the case of lapses) in athletes than in non-athletes, suggesting better allocation of resources to maintain selective attention and better attentional focus amid distraction [28,45].

Overall, our results seem to support the “cognitive component skills” theory, by showing a positive relationship between a regular sport participation and performance in the PVT. However, on the basis of the extant literature supporting the “selective improvement hypothesis” and the strong correlation between sport participation and fitness (i.e., our athletes were more fit than our non-athletes), we cannot discard the “selective improvement hypothesis” due to the low executive demands of the PVT used in the present study. Actually, the two hypotheses might not be mutually exclusive. In fact, we do consider the sport training context as a stimulating environment, where both cardiovascular fitness and perceptual-cognitive skills are enhanced, which might in turn influence cognitive function. It is important to note here, that our results do not distinguish between the impact of generic sports participation and the specific effects of soccer participation on vigilance performance. Further research comparing groups of participants from different sport types and with different levels of sport participation and cardiovascular fitness will be needed to clarify the specific, rather than combined, effect of both variables on vigilance performance.

Today’s lifestyle promotes sedentary activities in adolescents. The present study, together with previous research, point to a positive relationship between sport participation and cognitive functioning. Since the ability to sustain attention and cardiovascular fitness are critical for both school performance and health, respectively, this line of research, supported by our findings here, should encourage public health system administrators to implement policies aimed to increase adherence to sport practices during this important period of life.
